# The impact of the COVID-19 pandemic on higher education: Assessment of student performance in computer science

**DOI:** 10.1371/journal.pone.0305763

**Published:** 2024-08-14

**Authors:** Małgorzata Charytanowicz, Magdalena Zoła, Waldemar Suszyński

**Affiliations:** 1 Department of Computer Science, Lublin University of Technology, Lublin, Poland; 2 Systems Research Institute, Polish Academy of Sciences, Warsaw, Poland; Satyawati College (Eve.), University of Delhi, INDIA

## Abstract

The COVID-19 pandemic had radically changed higher education. The sudden transition to online teaching and learning exposed, however, some benefits by enhancing educational flexibility and digitization. The long-term effects of these changes are currently unknown, but a key question concerns their effect on student learning outcomes. This study aims to analyze the impact of the emergence of new models and teaching approaches on the academic performance of Computer Science students in the years 2019–2023. The COVID-19 pandemic created a natural experiment for comparisons in performance during in-person versus synchronous online and hybrid learning mode. We tracked changes in student achievements across the first two years of their engineering studies, using both basic (descriptive statistics, t-Student tests, Mann-Whitney test) and advanced statistical methods (Analysis of variance). The inquiry was conducted on 787 students of the Lublin University of Technology (Poland). Our findings indicated that first semester student scores were significantly higher when taught through online (13.77±2.77) and hybrid (13.7±2.86) approaches than through traditional in-person means as practiced before the pandemic (11.37±3.9, p-value < 0.05). Conversely, third semester student scores were significantly lower when taught through online (12.01±3.14) and hybrid (12.04±3.19) approaches than through traditional in-person means, after the pandemic (13.23±3.01, p-value < 0.05). However, the difference did not exceed 10% of a total score of 20 points. With regard to the statistical data, most of the questions were assessed as being difficult or appropriate, with adequate discrimination index, regardless of the learning mode. Based on the results, we conclude that we did not find clear evidence that pandemic disruption and online learning caused knowledge deficiencies. This critical situation increased students’ academic motivation. Moreover, we conclude that we have developed an effective digital platform for teaching and learning, as well as for a secure and fair student learning outcomes assessment.

## 1. Introduction

The COVID-19 pandemic brought with it a number of health, economic and social consequences. Indeed, the spread of the SARS-CoV-2 virus turned out to be so dangerous that many countries implemented new regulations in the educational field to limit physical contact. The pandemic-induced school shutdowns and sudden transition to remote teaching and learning at all levels of education. This change-over generated a number of technical and social problems [1–6]. These problems had also affected the academic community, although online or blended learning methods were implemented before the COVID-19 pandemic [7].

On March 12, 2020, a state of epidemic emergency was declared in Poland, and a week later–a state of pandemic. In consequence, the Minister of Science and Higher Education issued a regulation on the temporary suspension of the functioning of education institutes, lasting from March 12 till 25 2020 [8, 9]. On March 25, 2020, the education system, including higher education, was switched to online teaching and learning, as necessitated by the need to maintain social distancing measures. Universities had to adapt to the circumstances almost overnight. However, many universities were not fully prepared with regard to technical capabilities, educational resources and the skills of the teaching staff in organizing distance education [10–12]. Before the COVID-19 pandemic, the applicable regulations of the Ministry of Science and Higher Education did not encourage the authorities of most universities to invest in technologies for conducting fully remote studies. Poland was, however, not an exception in this respect. Many old, prestigious universities in Europe were also reserved about remote learning, and the virtual learning environment was mainly used as a teaching aid.

Fortunately, the information revolution had by this time developed more flexible approaches to learning with the form of Information and Communication Technology (ICT). Indeed, it is one of the leading factors that affect current teaching methodology [13–18]. E-learning systems, their accessibility and functionality, have provided new possibilities to acquire knowledge and to ease the burden of learning. As an outcome, remote teaching and learning are often seen as promising solutions that offer high flexibility and a learner-centered approach that enables students to learn at their own pace [19, 20]. Thus, the role of the teacher in the classroom has transformed from that of being the font of knowledge, to an instructional manager identifying relevant resources and creating collaborative learning opportunities. Moreover, online assessments have become increasingly important and now represent one of the most critical aspects of the educational process. Unfortunately, the role of ICT in higher education is still somewhat controversial.

The extreme situation caused by the COVID-19 pandemic provided an opportunity to revise our approach both to traditional and online learning, yet also posing challenges for the future of education systems. The main question of our research was whether the sudden transition to online teaching and learning caused by the COVID-19 pandemic had a negative impact on students academic performance and upon the reliability of the assessment process. We believe that our study can help to reduce the controversies related to remote learning and teaching.

## 2. Related works

Before the year 2020, the principal recipients of remote education were adults participating in professional development courses [21]. The COVID-19 pandemic outbreak, however, resulted in increased interest in methods of education that do not require physical meeting between students and teachers. The closure of educational institutions to mitigate the spread of COVID-19 compelled schools and universities to find alternative ways of continuing their operations. This led to the widespread adoption of online learning (e-learning).

The use of e-learning platforms has enabled the transformation of the traditional model of education in which the lecturer transmitted knowledge, into a model of supervised self-education. A separate line of research has been dedicated to the impact of remote education on university students, who are predominantly young adults, and, as such, are less subject to parental supervision. Topics under study include student attitudes towards distance learning [22, 23], the technologies and learning platforms utilized [24–26], and the impact of network quality on the smoothness of classes [22, 27].

A relatively well researched aspect of e-learning is the analysis of its advantages and disadvantages in comparison to traditional learning [28–30], including its application during the COVID-19 pandemic [31–34]. Undoubtedly, remote education has its benefits, among others, flexibility, speed, time savings [35, 36], as well as better use of the infrastructure and organizational savings for the institution [37]. Distance learning in the form of e-learning also comes with drawbacks, for example, limited interpersonal contacts [38], lack of immediate feedback [39, 40], and problems with self-discipline and adaptability [41–43]. Considering its strengths and weaknesses, e-learning can be viewed as either a replacement or augmentation of traditional approaches to education.

An integral part of remote education is the verification of its results. The topic was covered in literature in the pre-COVID era [44–46], but much less so during the pandemic [47, 48]. Our work focuses on the analysis of student performance under the e-learning setup during COVID-19 related confinement and afterwards. The differentiating characteristic of this paper is the fact that it covers a longer period of time, unlike some other research focusing only on a single academic semester [49].

The COVID-19 pandemic has provided the opportunity to advance usage of online platforms and digital media, as well as to create new education strategies. It should be noted that most students (and instructors) adapted successfully to online teaching and learning [50, 51]. However, certain studies [52–54] have indicated negative student feedback. In the year 2023, education has returned to more traditional teaching/learning approaches after more than two years of online learning.

The outbreak of COVID-19 presented a serious challenge to academic education by enforcing a drastic change in the teaching methods. For this reason, we formulated the following research questions:

1. How had the COVID-19 pandemic change applied teaching and learning strategies?
2. Did the COVID-19 pandemic have a disruptive effect on the academic performance of students resulting in knowledge deficiency?
3. How did the change from in-person to online learning affect the reliability of student assessment?

The rest of the paper is structured as follows. Section 3 presents the context of the study, materials and methods. Section 4 explains the results obtained. Sections 5 and 6 conclude our work and describe limitations and future scope.

## 3. Materials and methods

### 3.1. Design and context

The research was conducted in the Department of Computer Science of the Lublin University of Technology in Poland, the largest public technical university in the Lublin voivodship. This was a cross-sectional study carried out among students who were enrolled in the first semester of engineering studies in the academic years 2019/2020, 2020/2021 and 2021/2022 (from October to July). Because of the COVID-19 pandemic, the courses of interest in this study were conducted in different delivery formats (in-person, synchronous online and hybrid).

Traditional in-person course delivery format included lectures and laboratories. The former involved, primarily, oral presentations given to a group of students. A teacher-centered approach to learning was applied with discussion and multimedia presentation, as well as whiteboard or chalkboard visual aids to emphasize important points in the lecture. Moreover, a Learning Management System (Moodle LMS) was incorporated within the lectures to develop, organize, deliver and manage didactic materials and assess the effectiveness of education via tests, surveys or assignments. This tool was also employed to provide discussion forums. The faculty used the activity *Quiz* as a student self-assessment tool, as well as to determine knowledge and skills.

With regard to laboratory work, practical classes were conducted in programming laboratories for the selected courses. In such a teaching/learning format, we found that most students preferred working alone or conducting discussions with their partners or their neighbors.

All students used online manuals or didactic materials delivered by Moodle LMS. Final exams were held at the University via Moodle LMS through in-person proctoring, as this approach allowed the introduction of a live person to monitor the activity of students in a testing environment.

In the synchronous online course format, students obtained theoretical and practical education entirely online via Microsoft Teams by way of video meetings and Moodle LMS. Meetings in Teams include audio, video and screen sharing. All lectures were delivered synchronously using MS Teams. Practical sessions were conducted through online synchronous video meetings in small student groups. Interaction occurred via the discussion board, while MS Teams was also employed to enable scheduled online consultations. Supporting materials (videos, presentations, tasks to do, quizzes, and other didactic materials) were provided to the students through the Moodle LMS. Final exams were conducted under controlled conditions via Moodle LMS through online live proctoring by accepting screen, video and audio sharing.

The hybrid course delivery format combined in-person and online strategies. Students obtained theoretical education entirely online as synchronous sessions by way of MS Teams and Moodle LMS, whilst practical education was obtained through the traditional in-person format, in small student groups. Final exams were held at the University via Moodle LMS through in-person proctoring.

We analyzed exam scores across the first two years of the engineering studies using anonymous data from the Moodle. The Research Ethics Committee of Lublin University of Technology approved the study (Ethical Approval Reference: 3/2023).

### 3.2. Course selection

The following criteria were used to select the courses:

1. the courses covered algorithms and programming,
2. the courses had unchanged objectives and learning outcomes during the investigated period,
3. the courses were conducted by the same instructors using to the same tools and methods.

Two compulsory courses met these criteria: 1 –Introduction to Computer Science and 2 –Numerical Analysis Algorithms. Both courses were conducted in the Polish language and they provided fundamental knowledge for all areas of Computer Science learning and skills development. Enrolled students were obligated to complete 30 lesson hours of theory and 30 lesson hours of practical experience within a course length of 15 weeks. In the full-time option, four hours of classes were given each course week, and were distributed into two two-hour sessions. Herein, the first consisted of a master class lecture and the second consisted of an interactive problem-based learning laboratory. In the part-time option, the number of in-person teaching hours was reduced to half and classes were held, on average, twice a month, on Saturday and Sunday.

The Introduction to Computer Science course is taught in the first year and is covered in the first semester. Students who successfully completed the course gained five credits, according to the European Credit Transfer and Accumulation System (ECTS). The intention of the offered course is to provide students with knowledge of standard algorithms and data structures, and to provide them with the skills to analyze both the theoretical complexity of algorithms and their practical behaviors. The course covers the following topics:

- Introduction to algorithms and problem-solving techniques.
- Basic programming concepts, types, sequential data structures.
- Programming in Python.
- Searching and sorting algorithms.
- Examples of algorithms, algorithmic strategies.
- Testing and documenting programming code.
- Asymptotic notation and complexity analysis.
- Analyzing program code for correctness, efficiency, and errors.
- Automata theory and formal languages. Turing machine.
- Classes P and NP.

The knowledge and skills to implement and solve algorithmic problems using the mentioned algorithms are developed using Python.

The Numerical Analysis Algorithms course is taught in the second year and is covered in the third semester. Successful completion awards students with five credits, according to ECTS. The primary objective of the course is to develop basic understanding of numerical algorithms, as well as the skills to implement algorithms to solve computer-based mathematical problems. The course covers the following topics:

- Basic numerics, floating-point representation, convergence.
- Horner’s scheme.
- The theory of interpolation: Lagrange polynomial, Hermite interpolation, Neville’s iterative formula.
- Least square approximation.
- Numerical integration: Newton-Cotes formulas, Gaussian quadrature.
- Direct methods for solving systems of linear equations: Gaussian elimination, LU factorization, Cholesky decomposition.
- Householder method.
- Solving nonlinear equations and systems of nonlinear equations: Bisection method, fixed-point iteration, Newton’s method.
- Runge-Kutta methods for ordinary differential equations.
- Characteristic polynomial and eigenvalues.

The knowledge and skills to implement and solve algorithmic problems using the mentioned algorithms were developed using C++ due to its object-oriented programming with high performance, efficient memory management, low-level access to hardware and a rich standard library, including mathematical functions commonly used in numerical algorithms. These allow students to write efficient and customizable numerical algorithms. Objective C++ was one of the courses of the first year of studies.

### 3.3. The study participants

Study participants were selected from Computer Science students who were enrolled in the two mentioned compulsory courses: Introduction to Computer Science (ICS) (first semester) and Numerical Analysis Algorithms (NAA) (third semester). The first group of students began their studies in the academic year 2019/2020 in a traditional in-person course delivery format that was interrupted because of the confinement. They then continued their studies utilizing the synchronous online format. The second group consisted of students who began their studies in academic year 2020/2021 in the synchronous online format and continued these activities in a hybrid format. The third group of students began their studies in academic year 2021/2022 in a hybrid format that returned to an in-person format in the year 2022/2023. Online learning was supported by Moodle and MS Teams.

Only students enrolled in either the ICS and NAA courses participated in our research. Students who interrupted their studies and did not complete the courses were excluded. Thus, the study group included students who were enrolled in both courses and took both final exams. A total of 787 participants were selected. Table 1 summarizes the study participant groups according to education strategy.

**Table 1:** Student groups selection.

Student group	Sample size	Year of study commencement	Study strategy in the 1-st sem. (ICS)	Study strategy in the 3-rd sem. (NAA)
I	266	2019/2020	In-person	Online
II	300	2020/2021	Online	Hybrid
III	221	2021/2022	Hybrid	In-person

Males constituted 87.5% of the total study participants, while females constituted 12.5%. Regarding nationality, the majority, i.e. 85.5%, came from Poland, while 14.5% came from other countries, mainly Ukraine.

### 3.4. Online exam quizzes

In this study, the Moodle platform provided by the Computer Science Department from the Lublin University of Technology was applied to conduct the final exam process. Comparative analysis of student academic performance was anchored on the results obtained in their final exams. Final exams were carried through the Moodle platform using *Quiz activity*. All exams comprised questions of various types, including *Multiple Choice*, *Short Answer*, *Numerical* and *Essay* as follows:

- Multiple choice questions were employed for evaluating both theoretical and practical contents. For our purpose, the option *Multiple answers are allowed* was used. Multiple answers questions enable one or more answers to be chosen by providing check boxes next to the answers. We used a negative grade percentage for wrong answers, so that simply ticking all choices did not necessarily generate a full grade. If the sum of partial grades was negative, then the total grade for this question would be zero [55].
- Short answer or numerical questions were used to evaluate theoretical and practical contents. In a short answer question, the student types in a word or phrase in response to a question. This must exactly match one of the acceptable answers. Numerical questions resembled short-answer questions. Here, the difference was that numerical answers were allowed to have an accepted error for number.
- Essay questions were used to evaluate practical contents, mainly programming and coding skills. We employed essay-type questions to provide the option of answering by entering text online. The option *Require the student to enter text* was chosen. The *Response format* option was set to *Plain text*, *monospaced font* to improve the readability of code by ensuring consistent and clear alignment. This is particularly helpful for maintaining an organized layout. The essay questions had to be marked manually by the course instructor.

The number of multiple choice questions and short answer / numerical questions was comparable. One question was an essay question. Questions were created and stored separately in a Question bank and were organized into 10 categories according to the implemented curricula and learning outcomes. Each category consisted of at least 50 questions. Quiz settings were as follows:

- Quizzes included 20 questions worth 20 points. There were two categories of questions: theoretical and practical.
- Students were allowed to have one attempt at each quiz. The time limit option was set to 60 minutes.
- Students were not allowed to open other windows or programs while taking these quizzes.
- A password was required. The option *Block concurrent connections* was checked.
- The *Choose Sequential* navigation method was employed to compel the student to progress through the questions in order and not return to a previous question or skip to a later one.
- The timeframe when the students were able to see feedback was set to the option *After the quiz is closed* and the option *Whether correct* was checked.
- Employed questions were assessed for quality and modified for re-use in the next academic year.

Students were tested using the same evaluation methods and types of questions in in-person, synchronous online and hybrid groups. The Moodle platform collected assessment data and generated report statistics. The data containing students’ exam results (points) were collected and exported from the Moodle platform as.xlsx files.

### 3.5. Quiz report statistics

Quiz statistics provided test statistics and quiz structure analysis. The test statistics gave information on how students performed on a quiz, and employed descriptive statistics: average grade, median grade, standard deviation of grades, skewness and kurtosis. A detailed analysis of each question was given in quiz structure analysis, and applied the following measures: facility index, discrimination index and discriminative efficiency. Discriminative efficiency is a measure similar to discrimination index [55].

#### Facility index

In this work, facility index of a question was determined by the average score divided by the maximum score and represented as a percentage. A higher value indicated an easier question. The interpretation of its values is given in Table 2 [55].

**Table 2:** Facility index interpretation.

No	Facility index [%]	Interpretation
1	5 or less	Extremely difficult or something wrong with the question
2	6–10	Very difficult
3	11–20	Difficult
4	21–34	Moderately difficult
5	35–65	About right for the average student
6	66–80	Fairly easy
7	81–89	Easy
8	90–94	Very easy
9	95–100	Extremely easy

A high facility index (>80%) indicates the question was easy. A low facility index (<30%) indicates a difficult question.

#### Discrimination index

Discrimination index is the correlation between the score for this question and the score for the whole quiz represented as a percentage. If the score for the question and the score for the test are well correlated, the question can be categorized as a question with good discrimination. The maximum discrimination requires a facility index in the range 30%–70%, although this is not tantamount to high discrimination index. Discrimination index values should be interpreted according to Table 3 [55].

**Table 3:** Discrimination index interpretation.

No	Discrimination index [%]	Interpretation
1	50 and above	Very good discrimination
2	30–50	Adequate discrimination
3	20–29	Weak discrimination
4	0–19	Very weak discrimination
5	negative	Question probably invalid

A negative value of a discrimination index would mean that the best students got this question wrong more often than the worst students. A discrimination index of zero would mean it was a poor discriminator between good and bad students. Discrimination index is considered excellent when the value is higher than 40%, and considered good when it ranges from 20% to 40%.

#### Discriminative efficiency

The discriminative efficiency estimates how good the discrimination index is relative to the difficulty of the question. This attempts to discriminate between students of different ability, and the higher the value, the better is the question at discriminating between students of different abilities [55]. Values between 30%–50% provide adequate discrimination, while those above 50% provide very good discrimination.

### 3.6. Statistical analysis

Data collected was tabulated, and analysis was carried out by applying simple percentage analysis, as well as descriptive analysis, using mean, standard deviation and inferential analysis such as t-Student tests and ANOVA [56, 57]. We performed non-parametric alternatives such as a Mann-Whitney U test and the Kruskal-Wallis test to compare samples that cannot be assumed to be normally distributed [58, 59]. Statistical significance was set at p<0.05. Data analysis was performed using the Statistica Package, Version 13 (TIBCO Software Inc.).

## 4. Results

### Participants’ profile

Our study included 787 Computer Science students, aged 18 to 22 years. The participant background characteristics revealed that most students were male (87.5%) and native (Polish; 85.5%). Furthermore, most of the students were enrolled in full-time studies (85.5%) (Table 4).

**Table 4:** Background characteristics of computer science students.

Variable	Category	Frequency (%)
Gender	Female	98 (12.5)
	Male	689 (87.5)
Residency status	Native	673 (85.5)
	Non-native	114 (14.5)
Study option	Full-time	673 (85.5)
	Part-time	114 (14.5)
Study commencement year	2019/2020	266 (33.8)
	2020/2021	300 (38.1)
	2021/2022	221 (28.1)

The percentages of the students who began their studies in the academic years 2019/2020, 2020/2021 and 2021/2022 were comparable, around 30%. An important aspect of the analysis was the availability of data from the pre-pandemic period that was relevant for our investigations.

### Comparison of in-person, synchronous online and hybrid learning

The comparison of in-person, synchronous online, and hybrid teaching methods in student learning outcomes based on background characteristics is presented in Tables 5 and 6.

**Table 5:** Comparison of teaching methods in computer science student learning outcomes—As based on background characteristics: Introduction to computer science.

Variable		Introduction to Computer Science Mean ± Standard Deviation [points]			F	p-value
		in-person 2019/2020	online 2020/2021	hybrid 2021/2022		
Gender	Female	12.32±4.96	12.96±2.84	12.78±3.08	0.632	0.427
	Male	11.25±3.74	13.88±2.74	13.85±2.8		
	p-value	0.166	0.055	0.054		
	t	–1.39	1.925	1.937		
	df	264	298	219		
Residency status	Native	11.84±3.87	14.07±2.6	13.81±2.85	25.106	<0.001*
	Non-native	8.99±3.08	12.21±3.11	12.69±2.8		
	p-value	<0.001*	<0.001*	0.089		
	t	4.599	4.433	1.703		
	df	264	298	219		
Study option	Full-time	11.28±3.79	13.94±2.7	13.53±2.93	1.485	0.223
	Part-time	11.86±4.47	12.35±2.93	14.41±2.45		
	p-value	0.396	0.002*	0.071		
	t	–0.849	3.112	–1.814		
	df	264	298	219		

t–Independent Sample t-Test; F–Analysis of Variance;

*–p-value < 0.05.

**Table 6:** Comparison of teaching methods in computer science student learning outcomes—As based on background characteristics: Numerical analysis algorithms.

Variable		Numerical Analysis Algorithms Mean ± Standard deviation [points]			F	p-value
		online 2020/2021	hybrid 2021/2022	in-person 2022/2023		
Gender	Female	12.54±3.83	11.88±2.64	13.51±3.1	0.044	0.834
	Male	11.94±3.05	12.07±3.27	13.18±3.01		
	p-value	0.329	0.728	0.581		
	t	–0.978	0.349	–0.553		
	df	264	298	219		
Residency status	Native	12.2±3.19	12.33±2.99	13.42±2.94	15.754	<0.001*
	Non-native	11.01±2.73	10.58±3.79	11.34±3.13		
	p-value	0.022*	<0.001*	0.002*		
	t	2.306	3.573	3.063		
	df	264	298	219		
Study option	Full-time	12.21±3.16	12.12±3.19	13.14±3.01	2.01	0.157
	Part-time	10.82±2.76	11.38±3.19	13.57±3.1		
	p-value	0.011*	0.211	–0.831		
	t	2.57	1.253	0.407		
	df	264	298	219		

t–Independent Sample t-Test; F–Analysis of Variance;

*–p-value < 0.05.

The findings indicated that for the first semester course Introduction to Computer Science, the relation between learning outcomes and student gender was insignificant (p = 0.427). Moreover, the relation between learning outcomes and study option was also insignificant (p = 0.223). However, there was statistically significant difference between learning outcomes and residency status (p < 0.001). The findings indicated that during in-person and online studies, native students had significantly higher learning outcomes than did non-native students (p < 0.001). In addition, full-time students of online studies had significantly higher learning outcomes (p = 0.002) than did part-time students.

Regarding the learning outcomes of the students as obtained in the third semester course Numerical Analysis Algorithms, gender and study options were also insignificant (p = 0.834; p = 0.157) in relation to learning outcomes. In contrast, residency status was significant (p < 0.001). The findings indicate that native students had significantly higher learning outcomes than did non-native students (p < 0.001). Moreover, full-time students of online studies had significantly higher learning outcomes as compared to part-time students (p = 0.011).

The comparison of teaching methods in participant performance based on different semesters (courses) is presented in Table 7.

**Table 7:** Comparison of teaching methods in computer science student learning outcomes.

Course (semester)	Course delivery format Mean ± Standard deviation [points]			F	p-value
	in-person 2019/2020	online 2020/2021	hybrid 2021/2022		
Introduction to Computer Science (1-st semester)	11.37±3.9 ^a^	13.77±2.77	13.7±2.86	9.031	<0.001*
	**online** **2020/2021**	**hybrid** **2021/2022**	**in-person** **2022/2023**		
Numerical Analysis Algorithms (3-rd semester)	12.01±3.14	12.04±3.19	13.23±3.01 ^a^	4.117	0.017*

F–Analysis of Variance,

^a^–LSD post-hoc, significant differences between in-person and online, in-person and hybrid;

*–significant at p < 0.05

The differences in mean scores related to the first semester course Introduction to Computer Science, during online and hybrid studies, were significantly higher compared to in-person studies (LSD post-hoc, p < 0.001). However, mean scores related to the third semester course Numerical Analysis Algorithms, during online and hybrid studies, were significantly lower in comparison to in-person studies (LSD post-hoc, p < 0.001). Switching to traditional in-person studies in the academic year 2022/2023 did not degrade student performance.

### Quiz quality assessment

Tables 8 and 9 reveal the facility index, discrimination index and discriminative efficiency values from the final exams held from 2019/2020 to 2022/2023.

**Table 8:** Facility index, discrimination index and discriminative efficiency of the 1-st semester course final exams of introduction to computer science.

Course statistics	Course delivery format Mean ± Standard deviation		
	in-person 2019/2020	online 2020/2021	hybrid 2021/2022
Facility index	0.47±0.25	0.59±0.20	0.56±0.22
Discrimination index	0.34±0.17	0.37±0.20	0.37±0.14
Discriminative efficiency	0.47±0.24	0.48±0.26	0.52±0.21

**Table 9:** Facility index, discrimination index and discriminative efficiency of the 3-rd semester course final exams of numerical analysis algorithms.

Course statistics	Course delivery format Mean ± Standard deviation		
	online 2020/2021	hybrid 2021/2022	in-person 2022/2023
Facility index	0.51±0.25	0.52±0.34	0.55±0.32
Discrimination index	0.31±0.20	0.36±0.22	0.33±0.14
Discriminative efficiency	0.44±0.30	0.54±0.34	0.43±0.20

The lowest mean facility index was 47% ± 25%, while the highest mean facility index was 59% ± 20%. Moreover, the mean discrimination index was located within the range between 31% and 37% and the mean discriminative efficiency was found within the range between 43% and 54%. The results indicate, with regard to facility index, that most of the questions were moderately difficult, yet about right for the average student, and demonstrated adequate discrimination—regardless of the course delivery format.

## 5. Discussion and conclusions

In our study, we compared the learning outcomes of Computer Science students who were taught through synchronous online and hybrid systems, to those who learned in the traditional in-person system, and this revealed significantly higher learning outcomes when taught through online and hybrid systems versus in-person. It is worth noting that student scores showed an increasing trend in the years 2019–2023. Despite this, the significant difference in the results of the students’ final examination was not too large–as it did not exceed 10% of the maximal score.

A comparison between the student groups demonstrates that utilizing synchronous online learning can result in more enhanced educational opportunities for students. However, our findings indicated that native students had significantly higher learning outcomes than did non-native students. The reason could be that the study courses were held in Polish, which is a difficult language for non-native students to learn and utilize.

Several research studies have shown that online learning and the combination of online and in-person learning systems have positive and powerful roles in enhancing the effectiveness of education [19, 29, 41, 47, 60]. However, along with enhanced accessibility and flexibility, pure online learning also has several disadvantages, notably, the lack of interpersonal contacts and student satisfaction. In the hybrid form, however, flexibility and accessibility are enhanced, while human connection occurs.

Our results indicated that synchronous online learning could be appreciated as a successful method of conducting Computer Science education and can be used as a tool supporting traditional in-person methods. Although this approach is a little less flexible for teachers and students, and requires reliable technology, in comparison to asynchronous learning, this allows for more real time engagement and feedback [61].

As the effective measurement of knowledge acquired is an important component of Computer Science education, the use of the Moodle quizzes activity as a continuous assessment of students was analyzed according to statistical data such as the facility index, discrimination index and discriminative efficiency. Out of the exam tests conducted from the academic year 2019/2020 to 2022/2023, the mean facility index scores ranged from 47% to 59% and the mean discrimination index ranged from 31% to 37%. The statistic results indicated that, regarding facility index, most of the questions were moderately difficult and about right for the average student regardless of the course delivery format, and that a consistent and adequate level of discrimination indices was maintained. In addition, the similar results obtained in our study no matter the year, with three different groups of students, also confirmed the validity and reliability of the designed exam tests.

Although online learning requires extensive self-discipline, it allows universities to integrate new technologies into their offer, and hence, effectively facilitate the student learning process. After the COVID-19 pandemic, there has been a quick transition back to in-person teaching, but still there are many proffered activities being in an online format. At present, many students state that they prefer to learn through hybrid learning methods. Furthermore, several studies have shown that e-learning methods are used widely by students outside of their formal curricula for continuing their professional education [62]. This indicates that students and professionals appreciate and take advantage of self-paced learning environments in which they control their learning pace, information flow, selection of learning activities, as well as their time management. Thus, the digital transformation of the educational process has become a necessity to meet shifting student demands and seems to be one of the leading factors that affect current teaching methodology.

It is worth noting that the extreme situation caused by the COVID-19 pandemic provided an opportunity to revise our approach, both to traditional and online learning, but also posed challenges for the future of education systems. In conclusion, the results of the analysis allow us to answer the questions formulated before in the following way.

1. The COVID-19 confinement caused online education, which previously was mainly used as an addition to traditional learning methods, to become the mainstream, in particular, in Computer Science.
2. The COVID-19 pandemic did not have a disruptive effect that resulted in knowledge deficiency with regard to the academic performance of Computer Science students. In contrast, this situation increased student academic motivation. Indeed, students demonstrated higher exam scores during subsequent two academic years.
3. Despite the change from in-person to online learning, the reliability of student assessment remained at similar levels.

## 6. Limitations and future works

Our context is algorithms and programming in the first two years of the engineering studies program. While we believe that the long period under study is an advantage of this work, its limitation is the fact that it focuses only on the students of Computer Science. We based our research on the data comprising the performance of students in only two courses. Moreover, only the exam scores from the 1^st^ and 3^rd^ semesters were included in the study. The courses of other semesters were not assessed because they did not meet the required assumptions regarding the course selection. Another limitation of our study was that students could share information about the content of the exam. However, we randomly assigned students to subcategory sets to avoid sharing information. In the future it is worth considering extending the analysis to students of other fields, as well as take into account student performance in more courses.
